# Revealing the Electrophysiological Correlates of Working Memory-Load Effects in Symmetry Span Task With HHT Method

**DOI:** 10.3389/fpsyg.2019.00855

**Published:** 2019-04-24

**Authors:** Kai-Yu Chuang, Yi-Hsiu Chen, Prasad Balachandran, Wei-Kuang Liang, Chi-Hung Juan

**Affiliations:** ^1^Institute of Cognitive Neuroscience, National Central University, Taoyuan City, Taiwan; ^2^Taiwan International Graduate Program in Interdisciplinary Neuroscience, National Cheng Kung University and Academia Sinica, Taipei, Taiwan; ^3^Institute of Linguistics, Academia Sinica, Taipei, Taiwan; ^4^Brain Research Center, College of Health Science and Technology, National Central University, Taoyuan City, Taiwan

**Keywords:** complex span task, working memory-load, EEG, HHT (Hilbert-Huang transform), working memory

## Abstract

Complex span task is one of the commonly used cognitive tasks to evaluate an individual’s working memory capacity (WMC). It is a dual task consisting of a distractor subtask and a memory subtask. Though multiple studies have utilized complex span tasks, the electrophysiological correlates underlying the encoding and retrieval processes in working memory span task remain uninvestigated. One previous study that assessed electroencephalographic (EEG) measures utilizing complex span task found no significant difference between its working memory loads, a typical index observed in other working memory tasks (e.g., n-back task and digital span task). The following design constructs of the paradigm might have been the reason. (1) The fixed-time limit of the distractor subtask may have hindered the assessment of individual WMC precisely. (2) Employing a linear-system-favoring EEG data analysis method for a non-linear system such as the human brain. In the current study, the participants perform the Raven Advanced Progressive Matrices (RAMP) task on 1 day and the symmetry span (Sspan) task on the other. Prior to the formal Sspan task, the participants were instructed to judge 15 simple symmetry questions as quickly as possible. A participant-specific time-limit is chartered from these symmetry questions. The current study utilizes the Sspan task sequential to a distractor subtask. Instead of the fixed time-limit exercised in the previous study, the distractor subtask of the current study was equipped with the participant-specific time-limit obtained from the symmetry questions. This could provide a precise measure of individual WMC. This study investigates if the complex span task resonates EEG patterns similar to the other working memory tasks in terms of working memory-load by utilizing ensemble empirical mode decomposition (EEMD) of Hilbert-Huang transform (HHT). Prior expectations were to observe a decrement in the P300 component of event-related mode (ERM) and a decrement in the power of alpha and beta band frequency with increasing working memory-load. We observed a significantly higher P300 amplitude for the low-load condition compared to the high-load condition over the circumscribed brain network across F4 and C4 electrodes. Time–frequency analysis revealed a significant difference between the high- and low-load conditions at alpha and beta band over the frontal, central, and parietal channels. The results from our study demonstrate precise differences in EEG data pertaining to varied memory-load differences in the complex span task. Thus, assessing complex span tasks with the HHT-based analysis may aid in achieving a better signal to noise ratio and effect size for the results in working memory EEG studies.

## Introduction

In recent years, the individual differences in WMC effects on brain function has been an intriguing research question assessed by neuroscientists and psychologists likewise. How much can a person memorize an instantaneous information mainly depends on his/her WMC. Multiple facets of our daily lives relies on the length and breadth of this psychological construct. WMC remains to be the primary (though not unitary) descriptor of multitude of our everyday memory-related actions/habits down to a miniscule level. For instance, memorizing the digits of a phone number, number of items in the shopping list or recalling the names from a history class. All of these depends upon the individual’s WMC. Though few scientists argue a generalized value for the number of items that can be stored in WMC, there is no denying the existence individual differences at large. An individual’s WMC can be assessed using any of the multiple cognitive tasks such as the complex span task and n-back task to name a few (Broadway and Engle, 2011a,b; Dong et al., 2015).

Complex span task is one of the commonly used cognitive tasks that can evaluate individual WMC. Complex span task is a dual task that contains a distractor subtask and a memory subtask. Unlike other working memory tasks (e.g., n-back and Sternberg task) that are extensively used for electrophysiological studies, only a few studies have utilized complex span task to assess the neurophysiological data using EEG (Pesonen et al., 2007; Palomäki et al., 2012; Scharinger et al., 2015, 2017). Thus, it brings up the question of whether the complex span task shows any electrophysiological pattern similar to other widely used WM tasks by utilizing EEG. Previous studies have addressed the working memory load difference with the P300 component and the frequency band power of alpha and beta. Scharinger et al. (2015) discovered that with increasing n-back task levels, the P300 amplitude decreased at the Pz electrode. With an n-back task, Pesonen et al. (2007) discovered that the duration of event-related desynchronization response in the alpha frequency range (∼8–12 Hz) increased with increasing memory load and reaction time (for both, targets and non-targets). Also, longer duration of beta frequency range ERD response was observed with increasing memory load (Pesonen et al., 2007). With increasing memory load in the n-back task, Palomäki et al. (2012) observed an increase in the in the theta frequency range (∼4–8 Hz) spectral power with corresponding decrease in the 8–25 Hz frequency spectral power. Working memory load difference is a typical index observed in working memory task’s electrophysiological data. To the best of our knowledge, the only study discussing the complex span task observe no working memory load difference in P300 component (Scharinger et al., 2017). However, in the same study, the authors did report a much pronounced (though not significant) effect of the WM load on the P300 for the n-back task. We speculate that these results might be a consequence of the following instances. (1) The fixed time bound distractor subtask might not provide an appropriate way to examine individual WMC. (2) They utilized a linear-system favoring modality for analyzing their EEG data, which might not be appropriate to assess a complex non-linear system such as the human brain.

To investigate the electrophysiological data of the complex span tasks, we adopted the original automatic symmetry span (Sspan) task from Dr. Randall W. Engle’s lab^1^. In order to make our EEG results more precise, we applied ensemble empirical mode decomposition (EEMD), an improved and updated version of empirical mode decomposition (EMD, Huang et al., 1998). EMD is an acclaimed method utilized for analyzing the non-linear systems. In addition to EEMD, we also used event-related mode (ERM), an improved method for measuring ERP (Chang et al., 2016).

### Complex Span Task

Subsequent studies have helped us comprehend that WMC is the primary attribute of fluid intelligence (Gf) (Conway et al., 2003). These WMC measure obtained from any of the widely used cognitive tasks, such as Sternberg task, n-back task, digital span task and complex span task are related to fluid intelligence. Prior studies point out that each person’s working memory capacity (WMC) vary across a great range (Unsworth and Engle, 2007). In comparison to the other WM tasks, complex span tasks could be considered to be more robust primarily because, the WMC measures from the complex span tasks represent an adaptive system that aids not only in maintaining the task-relevant information accessible and active in memory but also allows additional information to be processed simultaneously (Conway et al., 2005). In the WM span tasks, the participants are required to assert their domain-specific skills like chunking of information, rehearsal and facilitating information storage whilst exercising domain-general capability that allows for executive attention and cognitive control. The demand on the executive function is typically evident when the participant has to shift from the memory subtask to the distractor subtask. Typically, this requires WM updating and to inhibit the previous task set’s stimuli whilst having to reject incorrect responses (Scharinger et al., 2017). Studies in agreement of the usage of complex span tasks argue that the complex cognitive behavior problem analysis and solving, reasoning, and comprehension could be better predicted using complex span tasks primarily because of the domain-general attention rather than its domain-specific demands (Scharinger et al., 2017; Engle, 2018). Compared to the simple span task, the complex span task suits as a better descriptor of fluid intelligence as it tests the individual’s ability to efficiently disengage from the most recently presented (attended) stimuli and no longer useful information (Engle, 2018). Hence, complex span task could provide a robust measure of WM capacity and account for individual differences efficiently.

In general, complex span tasks are dual tasks consisting any of the following tasks in combination. For instance, operation span task (Ospan) (Turner and Engle, 1989; Conway et al., 2005; Unsworth et al., 2005), reading span task (Rspan) (Daneman and Carpenter, 1980), Sspan task (Kane et al., 2004; Unsworth et al., 2009), and rotation span task (RotSpan) (Kane et al., 2004; Harrison et al., 2013). The task requires the participants to answer a simple question from a span task, following which they are required to memorize the displayed item sequentially. After several trials, participants need to recall the displayed items in the sequential order of their presentation.

For example, in the Ospan task, participants are presented with a simple mathematics problem alongside its answer. The participants are required to respond whether the correspondingly displayed answer is correct [e.g., (2 × 2) + 3 = 7?] or incorrect. Sequentially, the participants are required to memorize a certain item (e.g., words or letters). In the due course of the task, the math problem and item will appear several times. After each math-item sequence, participants were asked to recall the preceding items in the same sequential order. In this scenario, participants need to switch between two different subtasks, which requires working memory updating and inhibition of other irrelevant information. Hence, tapping into the neural evidence of complex span task behavior would prove to be a perfect resource to assess how instantaneous information processing and storage is carried out concurrently.

### Working Memory Load Difference in P300

Depending upon the tasks utilized for the course of the experiment, different neural correlates can be utilized to define the relevant results obtained. Such an index of working memory performance is the P300 component. It is defined as the positive component of around 250–500 ms post-stimulus onset (Johnson, 1993). It was first revealed by Sutton et al. (1965) and have been a common observation in the oddball paradigms (Squires et al., 1975). In the traditional oddball paradigm, participants received two kinds of stimuli. The standard stimuli and the target stimuli which amounts to 85% and 15% of the paradigm, respectively. The P300 amplitude was observed when the participants successfully discriminated the target stimuli from the standard stimuli. Furthermore, a larger P300 component was detected during the encoding phase for the stimuli that were successfully retrieved (Polich, 2007). This provides insight into indexing P300 as a correlate associated with attention and memory updation. Picton (1992) observed a decrease in P300 amplitude with corresponding increase in mismatch and/or task difficulty. Multitude of n-back task related studies validate that an increasing working memory load would in-turn lead to a corresponding decrease in P300 amplitude (Kok, 2001; Watter et al., 2001; Dong et al., 2015; Scharinger et al., 2015, 2017).

### Frequency Band Power of Working Memory Load Difference

Time–frequency studies suggest that working memory load is related to frequency band power such as alpha and beta band for the n-back task. Klimesch (2012) reported an increase in alpha oscillation when the participant pays more attention. Few studies report a decrease in the power of alpha and beta frequency with increasing working memory load (Pesonen et al., 2007; Scharinger et al., 2017). Contrary to this finding, in short-term memory task, studies show that oscillations in the alpha band increasing with short-term memory storage (Jensen, 2002). This contrast in findings undelays the primary aim of this study to assess the pattern of electrophysiological data in complex span tasks.

## Materials and Methods

Prior to the start of the experiment, participants received instructions regarding the study procedures and signed a written consent form informing participants about their right to withdraw from the experiment at any time. Participants had to perform the experiment on two different days. Raven Advanced Progressive Matrices (RAMP) task for about 30 min on 1 day and the Sspan task on the other with EEG being recorded for both the days.

### Participants

Forty healthy college students from National Central University consisting of 13 males and 21 females (mean age = 22.15 years, *SD* = 2.32, range: 20–28 years of age) participated in the experiment. All participants were right-handed and had normal or corrected to normal vision. Participants received a monetary reward for their participation upon successful completion of the experiment. The experimental procedures were carried out in accordance with the guidelines of the Research Ethics Office of National Taiwan University.

### Symmetry Span Task

The experimental task was adopted from Unsworth et al. (2005) and was divided into two sections. Prior to the formal experiment, participants needed to answer 15 simple symmetry questions. The participants were instructed to judge and respond whether the shape displayed is symmetrical along the vertical axis or not by clicking on a ‘yes’ or ‘no’ box as quickly as possible. After each question, the accuracy feedback was displayed to the participant. This section was implemented to facilitate a participant based time bound for the Sspan task and to eliminate the fixed time limit in the previous study (Scharinger et al., 2017). The time taken for each participant to answer the symmetry questions were quantified. This participant-specific time was utilized for thresholding the minimum display time for correct response. On successful completion of this section, the program was orchestrated to implement the minimum time for the correct response as the sum of average reaction time and 2.5 standard deviations for the formal (main) experiment. This varying (i.e., participant-specific) time based bound for the Sspan task will provide better measures in assessing the individual differences.

**FIGURE 1:**
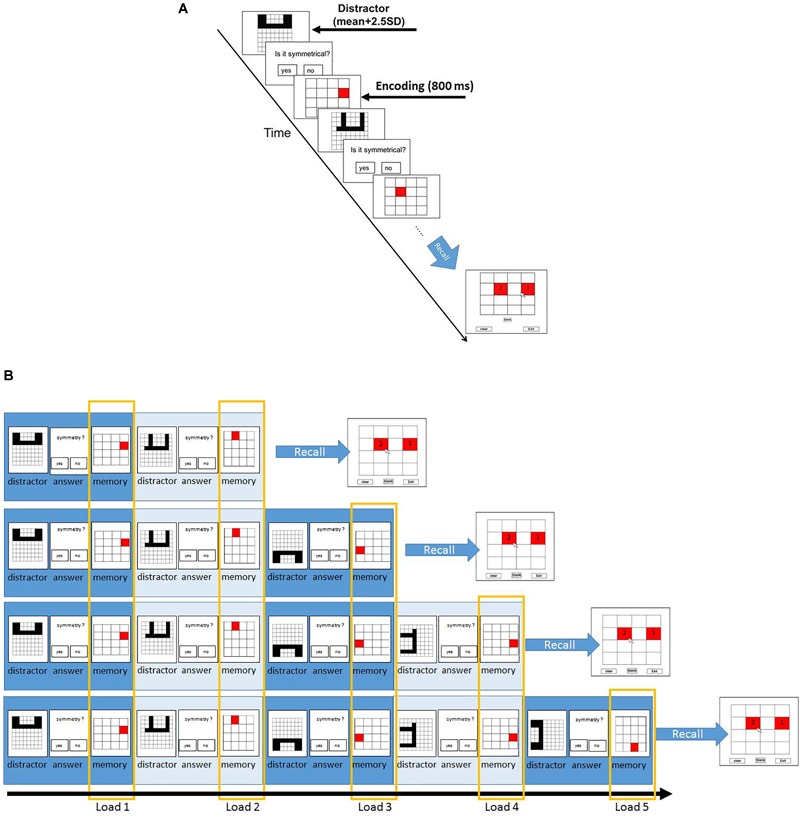
Paradigm and the different load conditions of symmetry span (Sspan) task. **(A)** Participants first judge whether the displayed shape is symmetrical along its vertical axis, followed by a red square appearing at one of the potential locations in a 4 × 4 grid. Participants then judge another displayed shape, followed by another red square. This symmetry-location sequence is randomly repeated from two to five times for each trial each time. After each symmetry-location sequence, participants were instructed to recall the red squares in the sequential order of their presentation. In order to assess the individual difference, the distractor subtask was designed to set the minimum time for correct response as the sum of average reaction time and 2.5 standard deviations in the formal (main) experiment. **(B)** The load conditions are defined according to their order of appearance. The first appearance of the symmetry-location sequence in each trial is categorized as load 1; the second appearance of the sequence is load 2 and so on.

Following this section, participants proceeded to the main experiment. In the main experiment, participants had to memorize the location of red squares in a 4 × 4 grid and solve some symmetry questions (Figure 1). Each trial started with a symmetry question. The time of presentation of each symmetry stimuli was the participant-specific time bound calculated using the previous symmetry section. Participants were required to judge if the presented stimulus was symmetrical along its vertical axis or not via button press. On solving the symmetry question, participants see a following red square at a different position. The squares were presented for 800 ms. The set size ranged from two to five. Each set size was repeated for ten times in random fashion. After the trials with the presentation of two to five squares and subsequent symmetry questions, the experiment proceeds to the recall phase. During the recall, participants saw a 4 × 4 grid and were required to report the location of the red squares presented during the trial in the sequential order of their presentation. To observe consistency in results, we only recruited participants who solved the symmetry questions with an accuracy of 85% and higher.

The Sspan score for each participant was calculated as the number of squares correctly recalled in their sequential order of presentation. The first square location that the participants had to memorize in each location sequence was regarded as load 1 condition; the second square location as load 2 condition, and so on. According to this rule, load 1 condition trials contained two-length location sequence to five-length location sequence whereas the load 5 condition trials contained only five-length location sequence. This made 40 trials for load 1 and load 2, 30 trials for load 3, 20 trials for load 4, 10 trials for load 5. To reach a sufficient number of trials for EEG analysis, we treated the combination of load 4 and load 5 as the high-load condition whereas load 2 was instituted as the low-load condition.

### EEG Protocol and Analysis

#### EEG Recording Parameters

Participants wore a 36-channel digital EEG cap (Quik-Cap) with Ag/AgCl sintered electrodes placed according to the international 10/20 system (FP1, FP2, F7, F3, Fz, F4, F8, FT7, FC3, FCz, FC4, FT8, T3, C3, Cz, C4, T4, TP7, CP3, CPZ, CP4, TP8, T5, P3, Pz, P4, T6, O1, Oz, O2, HEOL, HEOR, VEOU, VEOL, A1, A2). Offline was referenced to the left and right mastoid. All scalp EEG electrode impedances were kept below 5 kΩ. A Neuroscan amplifier (Nuamps) and Neuroscan 4.5 software were used for EEG acquisition of the signal digitized at a 1,000 Hz sampling rate. No band-pass filters were applied to the raw data in order to take most advantage of the EEMD method. We recorded the EEG signal simultaneously with the task and the signal analysis was performed observing the EEG epochs.

#### Event-Related Mode Analysis

**FIGURE 2:**
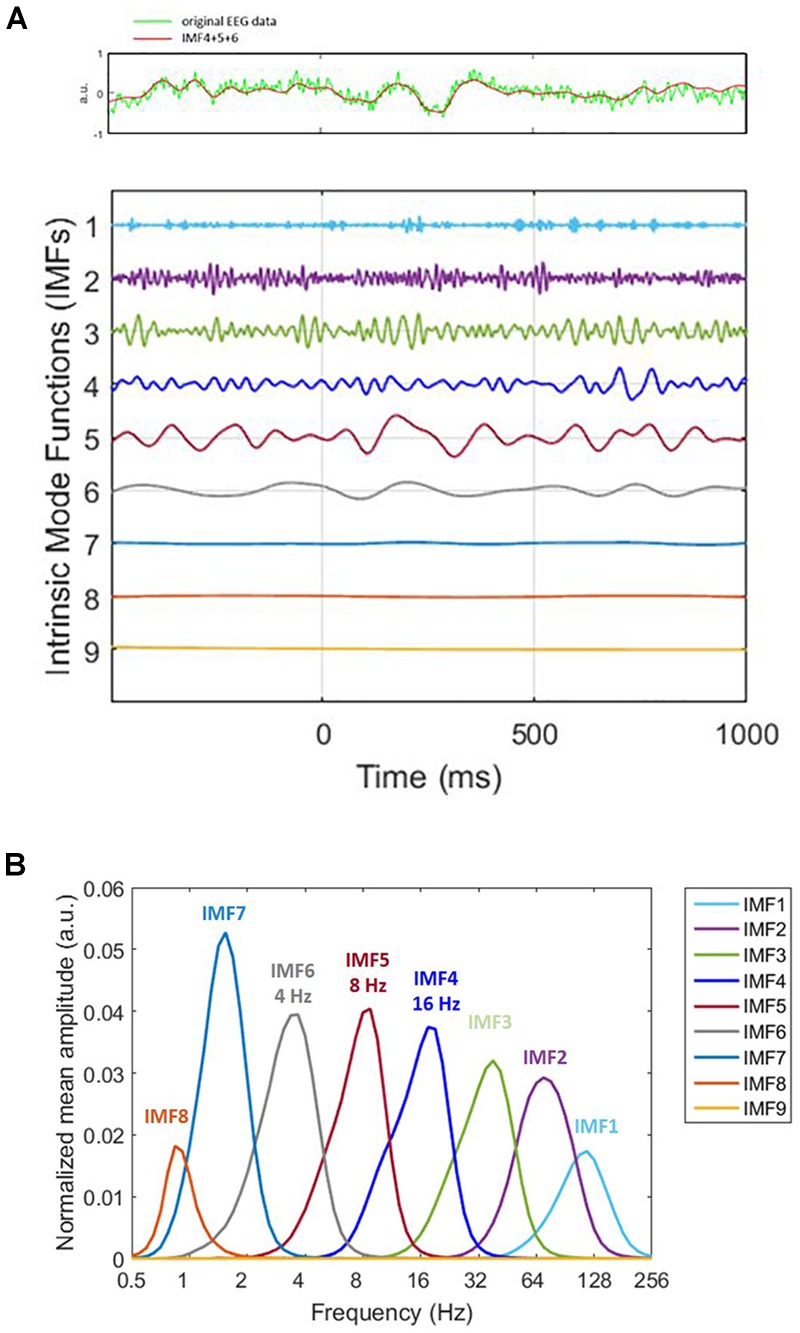
Decomposition of original signal. **(A)** On decomposing the original signal (the green line in the top panel) by utilizing ensemble empirical mode decomposition (EEMD), we obtained nine intrinsic mode functions (bottom panel). The *x*-axis represents time. 0 ms is the memory target onset. Different IMFs correspond to different physiological frequencies. For instance, IMF1 (1st IMF) corresponds to high-frequency noise, IMF2 and IMF3 correspond to gamma, IMF4 to beta. The frequencies we analyzed were alpha, beta, and theta, which were IMF4, IMF5, and IMF6. Therefore, we summed IMF4, IMF5, and IMF6 for ERM analysis (the red line in the top panel). The very low frequency is the trend. The time window for ERM analysis is –200 ms to 800 ms. **(B)** HHT amplitude spectrum marginal mean of each IMF. The peak of the distribution represents the frequency that accounts for the most portion of mean amplitude within the IMF.

To extract time–frequency information from the EEG signals, we applied HHT to the EEG data relative to the onset of memory target from -500 to 1,000 ms. Please note that the time window chosen for epoching is larger than the single-trial length (-200 ms to 800 ms). This was intentionally chosen to facilitate us to cover the trial boundaries with the additional data for the ERM and time–frequency analyses. HHT consists of Hilbert spectral analysis transform (Huang et al., 1998) and empirical mode decomposition (EMD). EMD sequentially decomposed a signal into the sum of a finite number of intrinsic mode functions IMFs) (Figure 2A). Two definitions were decomposed in each IMF. First, the number of zero-crossings, local maxima, and local minima must either be equal or differ at most by one. Second, the mean value of the envelope, which is defined by the local maxima and the local minima, should be zero. The IMFs represent different oscillatory modes contained in the data. To resolve the mode-mixing problem that might be caused by the original EMD method, we applied ensemble EMD (EEMD) (Huang et al., 2009), a noise-assisted version of EMD, for each trial. To apply EEMD, the IMFs were generated from ensemble means of trials by repeating EMD on the same signal with different sets of Gaussian noise (Figure 2B). The current EEMD analysis was applied with 100 ensembles. For each time of each ensemble, the amplitude of the Gaussian noise was 0.35% of the EEG segment’s standard deviation. The current analysis would focus on the IMF located in the alpha and beta band in order to facilitate the comparison with the current literatures. Hilbert spectrum was calculated for each trial and each IMF to acquire the instantaneous information about frequency and amplitude. The HHT was applied with customized MATLAB (Math Works) scripts with ensemble EMD code provided by the Research Center for Adaptive Data Analysis of National Central University, Taiwan. Further data processing and statistical analysis were performed using SPM8 for MEG/EEG (Wellcome Department of Cognitive Neurology, London, United Kingdom).

All epoched data were transformed by HHT. For Sspan data, we acquired nine IMF. The fourth, fifth, and sixth which corresponded to beta, alpha and theta wave correspondingly were selected and summated for the data we had analyzed. The epochs were averaged separately for each load of the trial. EEG epochs were analyzed from -500 ms prior to and 1,000 ms following the distractor onset. After epoching, independent component analysis (ICA) was performed to remove vertical eye blinks and was followed by artifact rejection with a ±100 μV threshold for every channel.

The P300 was quantified by subtracting the mean amplitudes of high-load condition electrodes from low-load condition electrodes. Analysis of P300 was confined to the electrodes at the frontal (F3, Fz, F4), central (C3, Cz, C4), and parietal (P3, Pz, P4) brain regions. The time window assessed for the P300 was between 300 and 450 ms. To verify the effect of working memory load difference, a paired *t*-test was performed.

## Results

### Behavioral Performance

**FIGURE 3:**
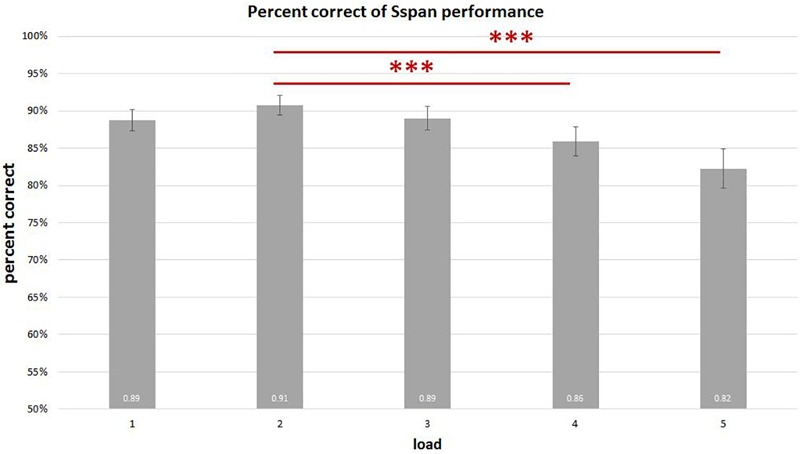
Sspan behavioral performance in each load. The results show a significant decrease in the correct percentage with corresponding increase in memory load. Load 1 and load 3 display no statistical difference [*F*(1.779,58.722) = 2.569, *p* = 0.091, $\eta_{p}^{2}$ = 0.07], whereas load 2 is significantly different from load 4 and load 5 [*F*(1.563,51.569) = 8.865, *p* = 0.001, $\eta_{p}^{2}$ = 0.21]. ^∗∗∗^Indicates *p* ≤ 0.001.

**FIGURE 4:**
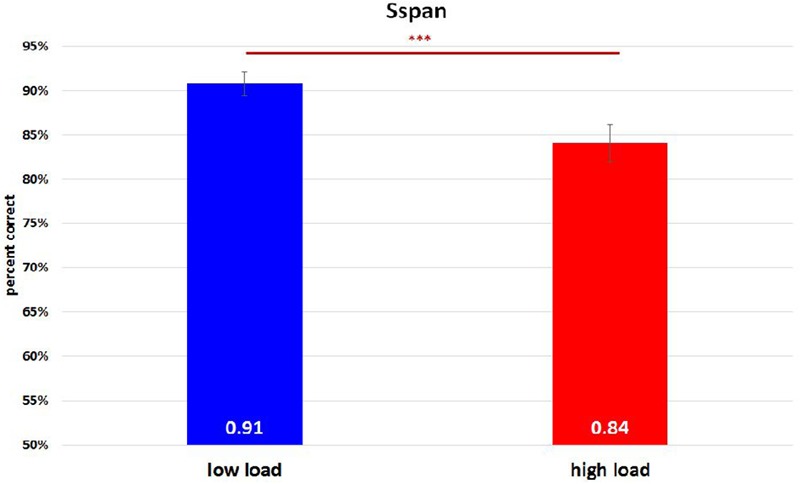
Sspan behavioral performance in high-load condition and low-load condition. The high-load condition was the combination of load 4 and load 5 whereas load 2 was regarded as the low-load condition. A paired *t*-test analysis revealed a significant difference between them [*t*(33) = 4.075, *p* < 0.000, *d* = 0.62]. ^∗∗∗^Indicates *p* ≤ 0.001.

On analyzing the correlation between RAMP task and Sspan task performance, we found that Sspan, positively correlated with the Raven task [*r*(32) = 0.37, *p* = 0.03]. Looking into the specifics of Sspan result, we considered the first position that participants need to memorize in each sequence as load 1, the second letter as load 2 and so on. Hence, we ended up with 40 trials for load 1 and load 2, 30 trials for load 3, 20 trials for load 4, 10 trials for load 5. After calculating each load’s correct percentage, a repeated-measure ANOVA with a Greenhouse–Geisser correction was performed to assess the load differences in behavioral data. The mean difference is significant at 0.5 level. A Bonferroni test was performed for the adjustment of multiple comparisons. The results (Figure 3) showed a significant decrease of correct percentage with increasing memory load [*F*(1.875,61.281) = 7.167, *p* = 0.002, $\eta_{p}^{2}$ = 0.18]. Load 1 and load 3 were statistically equal [load 1: 89%, load 2: 91%, load 3: 89%, *F*(1.779,58.722) = 2.569, *p* = 0.091, $\eta_{p}^{2}$ = 0.07] whereas a significant difference among load 2, load 4, and load 5 [load 2: 91%, load 4: 86%, load 5: 82%, *F*(1.563,51.569) = 8.865, *p* = 0.001, $\eta_{p}^{2}$ = 0.212] was observed. To reach the sufficient number of trials in EEG analysis, we combined load 4 and load 5 as high-load condition and load 2 as low-load condition. We quantified the effect size with Cohen’s *d* value. It is calculated as *d* = (M1 - M2)/s, where (M1 - M2) is the difference between the group means and s is the standard deviation of either group (Sullivan and Feinn, 2012). The two conditions also showed a significant difference with a medium effect size greater than 0.5 and lesser than 0.8 after paired *t*-test analysis [*t*(33) = 4.075, *p* < 0.000, *d* = 0.62] (Figure 4). High-load condition’s correct percentage (*M* = 0.84, *SD* = 0.134) was significantly lower to that of low-load condition’s (*M* = 0.91, *SD* = 0.089). The mean distractor time was 3,192 ms (*SD* = 1275, range: 1296–5643). The decreasing WM performance with increasing WM loads necessarily indicate that, higher WM loads demand higher cognitive facilitation and proportionately tax the WM resource. Also, with higher WM loads, fewer the individuals are able to memorize. Since the participants perform the Sspan task purely based on their own time gauge (participant-specific time limit), higher WM loads in our study testify the true degree of complexity for each participant.

### Event-Related Mode (ERM) Analysis

The ERM component we tested for assessing the WM load difference was P300. We chose the electrodes located in the frontal, central, and parietal brain areas (F3, Fz, F4, C3, Cz, C4, P3, Pz, P4).

**FIGURE 5:**
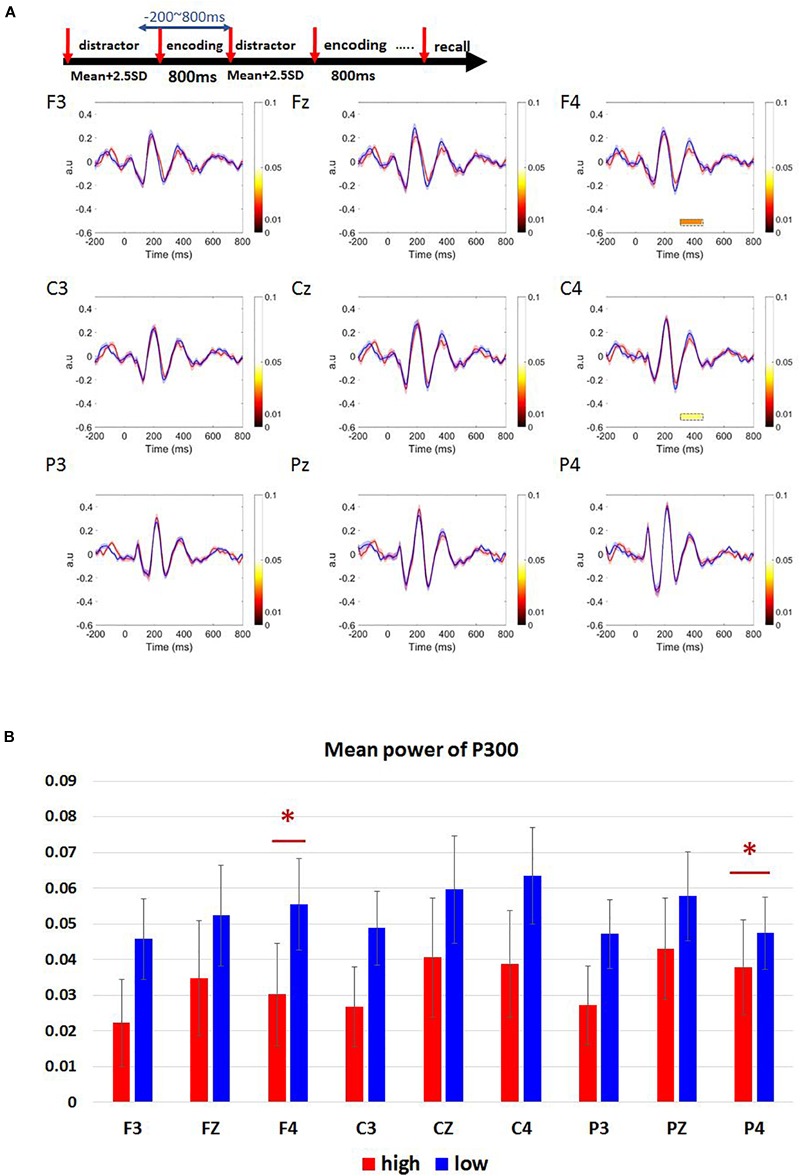
Results of ERM waveforms in Sspan P300 component of high- and low-load conditions. **(A)** P300 component was calculated as 300–450 ms post-stimulus onset. The blue line represents the low-load condition; the red line represents the high-load condition. P300 amplitude is higher for the low-load condition when compared to the high-load condition. We observe a statistical significance between the low and the high-load condition only over F4, C4 channels. **(B)** Mean power of P300 in frontal (F3, Fz, F4), central (C3, Cz, C4), and parietal (P3, Pz, P4) regions. The statistical significance was found over F4, C4 channels [F4: *t*(33) = –2.353, *p* = 0.025, *d* = 0.32, high-load: *M* = 0.03, *SD* = 0.08, low-load: *M* = 0.06, *SD* = 0.08; C4: *t*(33) = –2.144, *p* = 0.040, *d* = 0.30, high-load: *M* = 0.04, *SD* = 0.09, low-load: *M* = 0.06, *SD* = 0.08]. ^∗^Indicates *p* < 0.05.

We observed a positive peak around 300–450 ms after target onset in F3, Fz, F4, C3, Cz, C4, P3, Pz, P4 electrode channels (Figure 5A). A paired *t*-test was performed on the mean P300 amplitude. Low-load condition had higher P300 amplitude than high-load condition (Figure 5B). This trend was widespread in many electrodes whereas only the channels F4 and C4 showed statistical significance with an effect size (*d*) less than 0.2 [F4: *t*(33) = -2.353, *p* = 0.025, *d* = 0.32, high-load: *M* = 0.03, *SD* = 0.08, low-load: *M* = 0.06, *SD* = 0.08; C4: *t*(33) = -2.144, *p* = 0.040, *d* = 0.30, high-load: *M* = 0.04, *SD* = 0.09, low-load: *M* = 0.06, *SD* = 0.08].

### Time–Frequency

Using HHT, we reveal the time–frequency characteristics of attentional reorientation phenomenon. The purpose here was to examine whether participants could differentiate the conditions of complex span tasks by quantifying using EEG oscillations.

**FIGURE 6:**
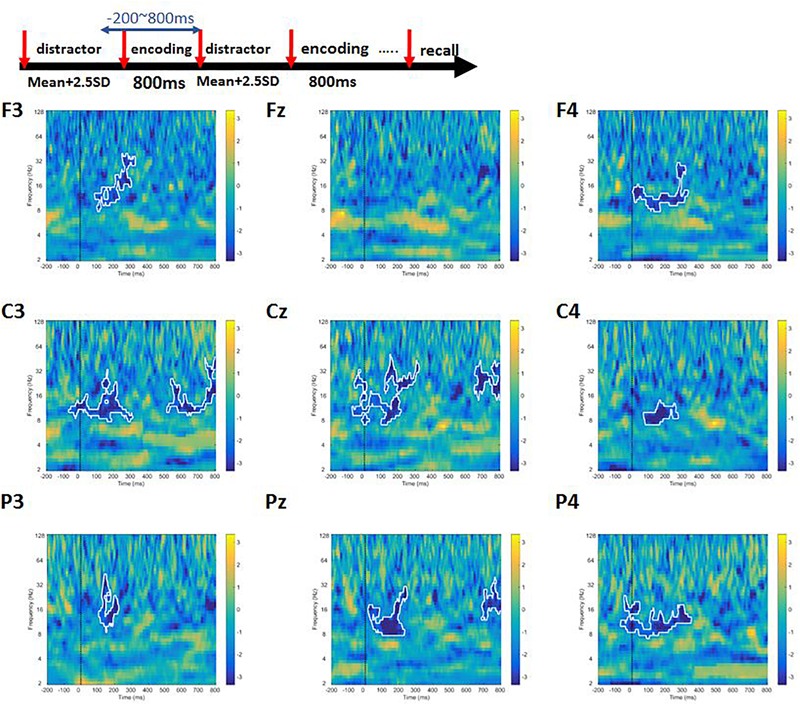
The contrast of high-load condition to the low-load condition of Sspan task. The area inside the white boundary line indicates *p* < 0.05 (cluster permutation). Alpha, beta, and gamma power was significantly higher in the low-load condition compared to the high-load condition.

A cluster permutation paired *t*-test was performed to find out whether there were difference between high-load and low-load conditions. We observed a significant difference between high- and low-load conditions at alpha and beta bands for frontal, central, and parietal channels (Figure 6). In addition, we noticed power decrement in high-frequency gamma band with increasing working memory load.

## Discussion

In the current study, we have analyzed the working memory EEG data for load difference in complex span tasks. Previous studies posit that the complex span tasks are positively correlated with fluid intelligence (Conway et al., 2003). The automated version tasks from Unsworth et al. (2005) were modified in MATLAB in order to acquire quality EEG data. When assessed, the Sspan score and RAMP score positively correlated. This validates that the MATLAB version of the complex span tasks is apt to assess the WMC. The Sspan performance scores were significantly different for the low-load and high-load condition with and effect size (*d*) of 0.62. This presents a medium effect size and thus emphasizing a reasonable significance for the difference in the WM load seen for the Sspan task in our study. According to prior studies, load difference have been assessed extensively in N-back tasks (Pesonen et al., 2007; Cong et al., 2009; Palomäki et al., 2012; Dong et al., 2015; Scharinger et al., 2015). However, the corresponding EEG data was not probed into. The previous complex span task study (Scharinger et al., 2017) with EEG measure finds no significant difference between working memory-loads. To our knowledge, our study is the first to report EEG evidence for a complex span task by HHT-based ERM and time–frequency analysis. This study discerns the working memory load difference in the complex span task. In summary, the Sspan task showed a significant decrease in the power of alpha and beta frequency with increasing working memory load levels whilst there is a decrease in P300 component with increasing working memory load levels. In the following sections, we will discuss the main outcomes in detail.

### Complex Span Task’s Load Difference Effect in P300 Component and Frequency Band Power

The present findings confirms the previous evidence (Kok, 2001; Watter et al., 2001; Dong et al., 2015; Scharinger et al., 2015, 2017) that the amplitude of P300 will significantly decrease for increasing working memory load. The prior study analyzed with band-pass filter finds no significant difference between working memory loads. In this study, we performed an ERM analysis to improve the signal-to-noise ratio. EEMD adaptively and locally decomposed any non-stationary signal in a sum of IMF that represent zero-mean, amplitude- and frequency-modulated components (Al-subari et al., 2015). We observe that the low-load condition has higher P300 amplitude than high-load condition in this version of the Sspan task. Although this trend is widespread across many electrodes, a significant difference in the P300 amplitude could be detected only at the F4, C3, C4, and P3 electrodes for the Sspan. The results from our study, in contrary to Scharinger et al. (2017), indicates the relevance of WM load on P300 at the C4 and F4 electrodes. We speculate that the utilization of a participant-specific time limit for the Sspan task and non-linear EEG analysis methods facilitated in bringing up the significance. We believe that the frontal-central brain network is sensitive to the processing of additional information simultaneously to chunking and rehearsing the WM stimuli presented. In Scharinger et al. (2017), the authors assessed the time–frequency representations (TFRs) by broadly classifying the electrodes considered into two regions (networks) namely FC1, FC2, FCZ, and CZ as the frontal-central and P3, P4, PZ, O1, and O2 as the parietal-occipital brain regions involved. A seemingly larger activity was seen for the frontal-central network relative to the parietal-occipital network. The results from the current study also express the same notion. P300 amplitude decreased with an increasing task difficulty (Kok, 2001) either by increasing load or by lowering the stimulus discriminability. Being distinguished from complex span task and n-back task, simple span task requires the participants to memorize a series of items, which may place fewer demands on working memory processes with relative to the other two tasks. This significance could be the possible reason why Scharinger et al. (2017) found relatively small differences in working memory load performance for digit span than n-back and complex span task. Although complex span task and n-back both require working memory processing, they might not probe the same working memory sub-component (Jaeggi et al., 2010). N-back is a single task where the participants need to memorize while comparing the stimuli. Whereas the complex span task is a dual task in which the participants need to shift between two different tasks. In addition, we speculate that the differences observed in Sspan could also have been caused by the conflicting/competing processes that take place between the two subtasks. In Sspan, the subtasks required the participants to judge whether the shape displayed is symmetrical or not and memorizing the location of red square in a 4 × 4 grid. Both subtasks were related to the visuospatial capacities. This might cause a competition of cognitive resources in this aspect that stands evident with the decrease the P300 in the higher load condition seen in our study. This pattern of result indicates that the working memory load differences in P300 component can also be found in complex span tasks by utilizing HHT-based analysis.

Prior studies claim that an increase in working memory load would lead to a decrease in the power of alpha and beta frequency (Pesonen et al., 2007; Scharinger et al., 2017). In line with these studies, the data from the current study shows a significant decrease in the power of alpha and beta frequency with increasing the working memory load in the Sspan task. Also, we observe a significant difference in the power of gamma band. It is strongly substantiated that the gamma power is associated with inhibition and working memory maintenance (Buzsáki and Wang, 2012; Roux et al., 2012). Therefore, we suggest that the observation of the decrement in gamma band in Sspan high-load condition may be an indication that the working memory can no longer be maintained in the high demanding condition or in high-load condition where the participants cannot inhibit the irrelevant information anymore. One previous study has also shown that adopting gamma-frequency transcranial alternating current stimulation (tACS) can affect visual working memory performance (Tseng et al., 2016). According to previous studies and our findings, we conclude that the working memory processing ability might decrease in high-load condition and lead to a lower power in the gamma band. Adhering to the P300 result, in Sspan task, the participants need to put more effort to resolve the conflict and interference that share the resource in location. More control is required to manipulate the perceptual representation and memory representation.

### Limitations of the Study

Although we find a difference in performance for different working memory load levels, not all of them show statistical significance. We speculate that this may be the consequence of insufficient number of trials. Due to the task procedure, the trial number decreases with an increase in memory load. In Sspan, for the first position, participants were required to memorize each position sequence and is duly regarded as the load 1 condition; the second position is regarded as load 2 condition, and so on. According to this rule, load 1 condition ranged between two-length position sequence to five-length position sequence whereas the load 5 condition consists of five-length position sequences only. Even though we combine loads 4 and 5 conditions to procure the high-load condition, we regard that the trial number might not be sufficient enough to show statistical significance. Future work could be aimed at facilitating an increase in the trial number of large letter sequence (e.g., four-length position sequence and five- position letter sequence) whilst keeping the trial number of small letter sequence same as the current experiment. We believe this prevents the task from being too lengthy.

## Conclusion

The current study aims to find if the complex span tasks show working memory load difference in EEG data by utilizing EEMD of HHT. Our results show a significant decrease in P300 component and the power of alpha and beta frequency with increasing working memory load levels. This indicates that complex span tasks share P300 component and powers of alpha and beta frequency patterns similar to the other working memory tasks such as n-back tasks. Thus, utilizing complex span tasks with the HHT-based analysis may aid in attaining a better effect size and signal to noise ratio for the results in working memory EEG studies. Moreover, we deduce that the significant load difference in EEG data may also have been the consequence of the competition between the two subtasks in the complex span tasks. When the two subtasks in complex span task are relevant, more working memory processing ability is required for the task. As the processing load increases, the working memory might no longer be well-maintained, thereby leading to a decrease in both P300 amplitude and gamma power. Also, we speculate that the load difference observed in the EEG data might also be an effect of the resource competition between the two subtasks. In order to clarify this issue, our future work will focus on combining Ospan and Sspan tasks, i.e., Ospan’s distractor subtask with Sspan’s memory subtask or Sspan’s distractor subtask with Ospan’s memory subtask.

## Ethics Statement

This study was carried out in accordance with the recommendations of the Research Ethics Office of National Taiwan University, Taipei, Taiwan. All subjects gave written informed consent before their participation in the experiments.

## Author Contributions

C-HJ, K-YC, and Y-HC designed the experiments. K-YC and Y-HC collected the data. K-YC, Y-HC, C-HJ, and W-KL analyzed the data. K-YC, Y-HC, C-HJ, and PB wrote the manuscript.

## Conflict of Interest Statement

The authors declare that the research was conducted in the absence of any commercial or financial relationships that could be construed as a potential conflict of interest.

## Footnotes

1 http://englelab.gatech.edu/index.html
